# Relationship between Chronic Short Sleep Duration and Childhood Body Mass Index: A School-Based Cross-Sectional Study

**DOI:** 10.1371/journal.pone.0066680

**Published:** 2013-06-21

**Authors:** Claudia Pileggi, Francesca Lotito, Aida Bianco, Carmelo G. A. Nobile, Maria Pavia

**Affiliations:** Department of Health Sciences, University of Catanzaro “Magna Græcia”, Catanzaro, Italy; Simon Fraser University, Canada

## Abstract

**Objective:**

To assess relationship between obesity and chronic shorter sleep duration in children and to determine if lack of sleep represents an independent determinant of childhood Body Mass Index.

**Methods:**

This cross-sectional study was conducted in all children enrolled in the fifth class (approximately 10 years of age) of all public primary schools in Catanzaro (Southern Italy). The overall response rate was 62% resulting in 542 participating children. Parents completed a questionnaire with information on their demographics and socio-economic characteristics, their health status, characteristics of their child birth and health status. The sleeping habits were investigated in the 3 months preceding the consultation and parents were asked to indicate hours of bedtime and wake-up of their children. Multivariate linear regression analysis was performed to examine the association between child BMI and chronic lack of sleep.

**Results:**

36.7% of the children surveyed were overweight or obese. A quarter of children did not routinely play sports and many of them spent more than an hour a day watching TV (60.7%) and using videogames or computer (51.1%). Widespread dietary habits were inadequate, especially concerning vegetables and fruit intake with more than 95% of children who consumed insufficient amounts. The average duration of sleep was equal to 9.4 (SD = ±0.6) hours, and the short-sleepers accounted for 38.9% of the total sample. The results of multivariate analysis showed a significant 0.77 Kg/m^2^ increase of BMI for children classified as short compared to normal sleepers (95%CI = 0.16–1.38, p = 0.01).

**Conclusions:**

Chronic lack of sleep appears to be associated to higher BMI even in middle childhood and strongly suggests that public health strategies, focused on promoting healthy lifestyles should include an innovative approach to ensure an adequate duration of sleep at night especially in children, alongside more traditional approaches.

## Introduction

In recent decades childhood prevalence of overweight and obesity increased worldwide from 4.2% in 1990 to 6.7% in 2010, and this trend is expected to reach 9.1% in 2020 [1]. These findings are particularly alarming since childhood obesity is strongly associated with a wide range of serious health complications and an increased risk of premature illness and death later in life [2], [3].

As well as obesity and overweight, a further “epidemic” has spread in recent years, following the progressive chronic sleep deprivation in the population. Data from the National Sleep Foundation show that, from 1998 to 2005, the number of USA adults that sleep an average of 8 hours per night has decreased from 35% to 26% [4]. Scientific debate has recently focused on the association between the reduction of sleep duration and obesity among children and several recent reviews, conducted to evaluate the consistency of evidence supporting a relationship between lack of sleep and obesity in different age groups, have shown that sleep deprivation is an independent risk factor for obesity and overweight [5], [6].

Despite several public health interventions that have been carried out for many years to try to reduce the prevalence of obesity, the phenomenon is still growing. Therefore, it is necessary to identify innovative approaches, based on a clear understanding of the emerging determinants to develop preventive strategies that should complement those already established.

Relationship between obesity and chronic shorter sleep duration has undoubted relevance in terms of public health implications, since the latter represents a potentially modifiable risk factor, and can be a target of prevention campaigns for the promotion of healthy lifestyles. To date, the link between the two conditions, even though it finds support in the results of several studies, is still controversial especially in the intermediate age. We focused our interests on this specific age group and carried out a study in a sample of Southern Italian children around 10 years old, with the aim to assess the role of characteristics of sleep as an independent determinant of BMI.

## Methods

The study was approved by the Institutional Ethical Committee (‘Mater Domini’ Hospital of Catanzaro, Italy, study number 2010/8).

This cross-sectional study was conducted on the children enrolled in the fifth class (approximately 10 years of age) of all public primary schools in Catanzaro (Southern Italy) during a one year period (2010–2011). The involvement of the subjects in the study took place with the written consent of their parents. Parents completed a questionnaire with information on their demographics and socio-economic characteristics, their health status (height, weight, occurrence of chronic diseases, smoking habits), characteristics of their child birth (gestational age, birth weight, breastfeeding) and health status (occurrence of diseases, surgical interventions, chronic use of drugs). For the assessment of children’s sleep characteristics, according to the procedure of Iglowstein and colleagues [7], parents also answered various questions on bedtime, wake time and eventual daytime napping, distinguishing the pattern of sleep on school days and over the weekend. The sleeping habits were investigated in the 3 months preceding the consultation and parents were asked to indicate hours of bedtime and wake-up of their children. Sleep duration was calculated as the average number of hours slept on weeknights and on weekend nights. We did not include eventual napping habits because, as previously reported [7], only a negligible percentage of children over 7 years old habitually sleep during daytime. Duration of sleep was classified according to the percentiles for nighttime sleep duration for ages, defined by the Second Zurich Longitudinal Study and Generational Study [7] and “short sleepers” were defined as children that slept an average of nighttime hours/day less than or equal to the 10^th^ percentile for age.

All students participating in the study completed a questionnaire administered by face-to-face trained interviewers focusing on health behaviours related to obesity, such as physical activity, diet, time spent watching TV and playing videogames or using computer. Frequency of sports activities was investigated as the number of times per week spent practicing sports or getting exercise in the previous 12 months; school-related activities such as physical education were included in the count. Food habits were recorded as self reported of food groups referring to the food pyramid and taking into account both school and home dietary habits. Appropriate number of daily servings of food groups was calculated according to the Nutrition Guidelines provided by the Italian Institute of Nutrition [8]. Dietary habits were considered inadequate if the number of servings of each food group consumed was higher or lower than the recommended, since each food group cannot be interchanged with those of another and none of the food group is more important than another [8].

Examinations were conducted at school by trained examiners and then calibrated. Height and weight were recorded on bare-footed subjects, and waist circumference was measured at the umbilicus level over the T-shirt.BMI was calculated by dividing weight (in kg) by the square of height (in m). Subjects were categorized as “overweight” or “obese”, using the tables of BMI percentiles developed for 6 to 20 years of age subjects by the Italian Pediatrics Society of Diabetology and Endocrinology (SIEDP) [9]. These tables provide the percentiles of growth not only in relation to age and sex, but also to geographic area. We used the percentiles for Southern Italy.

### Statistical Analysis

Data were summarized using frequencies and percentages for categorical data and mean and standard deviations for continuous data. In the primary analysis, we performed univariate regression models to examine the association between BMI and several explanatory variables, that are established on proposed risk factors of childhood obesity. Then we developed a multivariate linear regression analysis. Child BMI was the outcome variable in all models and the independent variables included were the following: gender (0 = male, 1 = female), age (continuous), maternal BMI (0 = low/normal weight; 1 = overweight/obesity), parental education’s highest level (continuous, in years), breastfeeding at least until the 3^rd^ month (0 = no, 1 = yes), birthweight (continuous, in kilograms); daily servings of each food group (cereal: continuous; vegetable and fruit: continuous; milk and derived: continuous; meat and fish: continuous; snack: continuous), watching TV (three categories: 1 = <1 hour/day; 2 = 1–3 hours/day; 3 = >3 hours/day), playing videogames or computer use (four categories: 0 = never; 1 = <1 hour/day; 2 = 1–3 hours/day; 3 = >3 hours/day), physical activity (three categories: 0 = never; 1 = 1–3 days/week; 2 = >3 days/week). As regards to sleep duration, it was investigated as nighttime sleep duration (0 =  normal sleepers, 1 =  short sleepers), nighttime sleep duration (continuous) and as the difference between weekend and weekday sleep duration (continuous). We did not include pubertal status among the variables included in the model because only 3.7% of children had onset puberty. Regression coefficient (β), standard deviations (SD) and 95% confidence intervals (CIs) were calculated.

Finally, we also modeled overweight/obesity as the outcome variable of the logistic regression analysis, and results are presented as odds ratios (ORs) and 95%CIs. All reported P values are two-tailed. The data were analyzed using the Stata software program, version 11 (Stata Corporation. College Station, TX).

## Results

The overall response rate was 62% resulting in 542 participating children. The main characteristics of the examined sample are reported in Table 1. Students were evenly distributed among males and females, with a mean age of 9.9 (SD = ±0.4) years (range 9–11). The mean BMI of the study population was 19.45 (SD = ±3.4) and, according to the tables of percentiles, 36.7% of the children surveyed were overweight or obese with a prevalence of overweight/obesity higher in males (41.3%) than females (31.8%); moreover 65% of fathers and 30.5% of mothers showed overweight and obesity. Regarding life-style habits, a quarter of children did not routinely play sports and many of them spent more than an hour a day watching TV (60.7%) and playing videogames or using computer (51.1%). Widespread dietary habits were inadequate, especially concerning vegetables and fruit intake with more than 95% of children who consumed insufficient amounts, but also in respect to cereals and milk groups, daily consumptions were adequate only in 33.5% and 29.5% of children, respectively (data not shown).

**Table 1:** Selected characteristics of the study population and results of univariate and multivariate analyses relating several variables to BMI.

			Univariate model	Multivariate model^a^
Variable	N (% ) or Mean±DS	BMI^b^ (Mean±DS)	Regression coefficient; SE (*p,* 95%CI)	Regression coefficient; SE (*p,* 95%CI)
**Sex**				
*Female*	261 (48.1)	19.36±3.3	*Reference category*	–
*Male*	281 (51.9)	19.53±3.5	−0.17; 0.29 (*0.55,* −0.74;0.4)	−0.34; 0.31 (*0.27,* −0.94;0.26)
**Age, years**	9.9±0.4	19.54±3.4^c^	−0.40; 0.32 (*0.22,* −1.04;0.24)	−0.24; 0.35 (*0.49,* −0.94;0.45)
**Parental education, years** d	13.2±3.6	19.54±3.5^e^	−0.005; 0.04 (*0.91,* −0.08;0.07)	0.02; 0.04 (*0.63,* −0.07;0.11)
**Maternal BMI** d				
*Low/Normal weight*	354 (69.6)	19.03±3.1	*Reference category*	–
*Overweight/Obesity*	155 (30.4)	20.5±3.8	1.42;0.32 (*<0.001,* 0.78;2.04)	1.4; 0.33 (*<0.001;* 0.75;2.06)
**Breastfeeding (at least until 3^th^ month)** d				
*Yes*	401 (76)	19.46±3.4	Reference category	*–*
*No*	127 (24)	19.47±3.4	−0.01; 0.35 (*0.99,* −0.69;0.68)	−0.02; 0.36 (*0.96,* −0.72;0.68)
**Birth weight, kilograms** d	3.2±0.5	21.3±4.1^f^	0.69; 0.28 (*0.01,* 0.14;1.24)	0.59; 0.29 (*0.04,* 0.02;1.16)
**Dietary habit** g				
**Cereal group, daily servings**	4.1±1.5	19.1±3.3	0.28; 0.10 (*0.005,* 0.08;0.47)	0.19; 0.10 *(0.07*, 0.02;0.39)
*Vegetable and fruit group, daily servings*	2.3±1	18.41±3	0.09; 0.14 (*0.53*, −0.18;0.36)	0.11, 0.15 (*0.45,* −0.18;0.41)
*Milk group, daily servings*	1.6±0.7	19.48±3.4	0.14; 0.20 (*0.49,* −0.26;0.53)	0.16; 0.22 (*0.47,* −0.27;0.59)
*Meat/fish group, daily servings*	1.1±0.5	19.57±3.5	0.49; 0.29 (*0.09,* −0.08;1.05)	0.46; 0.31 (*0.14,* −0.16;1.08)
*Snack group, daily servings*	1.4±1.1	19.65±3.5	−0.26; 0.13 (*0.04,* −0.51; −0.01)	−0.29; 0.14 (*0.04,* −0.56; −0.02)
**Watching TV, hours/day**				−0.21; 0.27 (*0.43,* −0.74;0.31)
*<1*	213 (39.3)	19.49±3.4	*Reference category*	*–*
*1–3*	308 (56.8)	19.44±3.4	−0.04; 0.30 (*0.9,* −0.63;0.56)	
*>3*	21 (3.9)	19.25±3	−0.23; 0.78 (*0.76,* −1.76;1.29)	
**Computer/videogame use, hours/day**				0.20; 0.21 (*0.34,* −0.21;0.62)
*Never*	116 (21.4)	19.41±3.8	0.003; 0.38 (*0.99,* −0.73;0.74)	
*<1*	277 (51.1)	19.40±3.3	*Reference category*	*–*
*1–3*	141 (26)	19.58±3.4	0.18; 0.35 (*0.60,* −0.51;0.87)	
*>3*	8 (1.5)	19.49±3.4	0.09; 1.22 (*0.94,* −2.3;2.48)	
**Physical activity, times/week**				−0.44; 0.25 (*0.08;* −0.92;0.04)
*Never*	136 (25.1)	19.61±3.8	*Reference category*	*–*
*1–3*	315 (58.1)	19.66±3.3	0.06; 0.35 (*0.86,* −0.62;0.74)	
*>3*	91 (16.8)	18.5±3	−1.1; 0.46 (*0.02,* −1.1; −0.2)	
**Duration of nighttime sleep, percentile** d				
*Normal sleepers*	330 (61.1)	19.1±3.3	*Reference category*	–
*Short sleepers*	210 (38.9)	20.01±3.5	0.92; 0.30 (*0.002,* 0.34;1.5)	0.77; 0.31 (*0.01,* 0.16;1.38)

a N = 496 subjects after exclusion of participants with missing data.

b The mean and standard deviation (SD) of BMI of the sample is 19.45±3.4.

c referred to the age of 10 years.

d the number that do not add to 542 are due to missing data for the variable.

e referred to the parental education of 13 years.

f referred to birth weight ≥4 kilograms.

g referred to recommended daily servings.

The average duration of sleep was equal to 9.4 (SD = ±0.6) hours, and short sleepers accounted for 38.9% of the total sample. Almost 60% of the children went to sleep between about 9∶30 p.m. and 10∶00 p.m. on weeknights. Moreover, children were very likely to change their sleep pattern on weekends with a mean of hours slept during weekend nights of 10.2±0.9 compared to 9.3±0.6 hours in the weekday nights (data not shown).

The results of the univariate analysis indicated that children higher BMI was significantly associated with maternal overweight and obesity (β = 1.42, 95%CI = 0.78–2.04, p<0.001) and higher birth weight (β = 0.69, 95%CI = 0.14–1.24, p = 0.01). Highlights of the analysis of the eating behaviours and physical activity were: each additional daily serving in the cereal group was significantly associated with a 0.28 (95%CI = 0.08–0.47, p = 0.005) increase in BMI, while an inverse association was noted with snacks intake (β = −0.26, 95%CI = −0.51–0.01, p = 0.04) and physical activity performed at least three times a week (β = −1.1, 95%CI = −1.1–0.2, p = 0.02). Regarding sleep duration, univariate regression model has shown a statistically significant higher BMI (β = 0.92, 95%CI = 0.34–1.5, p = 0.002) in short compared to normal sleepers.

**Figure 1:**
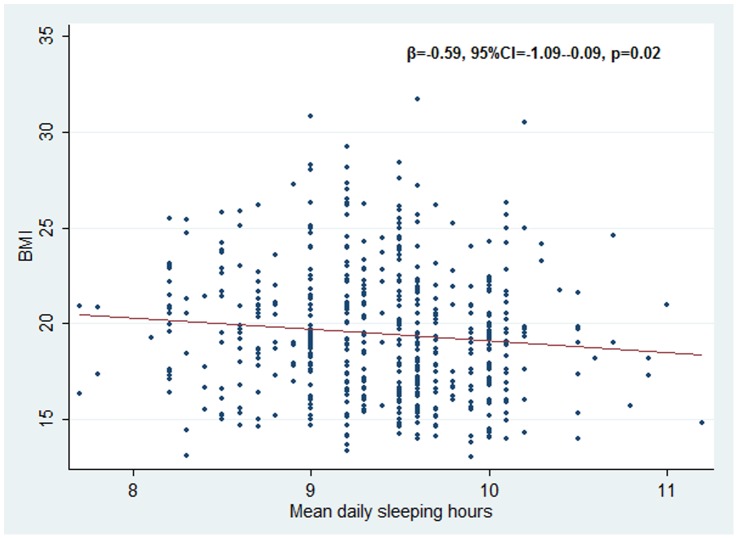
BMI scatterplot and regression line according to mean daily sleeping hours.

This statistically significant association was confirmed testing the sleep duration as continuous variable (β = −0.59, 95%CI = −1.09–0.09, p = 0.02) as showed in the Figure 1, whereas it was not confirmed when sleep deprivation was measured through the difference between weekend and weekdays mean hours of sleep (β = −0.1, 95%CI = −0.4–0.19, p = 0.51).

When the multivariate regression analysis was performed, the results basically did not change. Indeed, a significant 1.4 Kg/m^2^ increase of BMI was found for children whose mother was overweight or obese (95%CI = 0.75–2.06, p<0.001) and BMI raised of 0.59 Kg/m^2^ for each additional kilogram of child’s birth weight (95%CI = 0.02–1.16, p = 0.04). Furthermore, higher BMI was significantly associated with a lower daily consumption of snacks (β = −0.29, 95%CI = −0.56–0.02, p = 0.04). Adjustment for explanatory variables did not substantially change the relation between children BMI and sleep pattern that was associated with a 0.77 Kg/m^2^ increase in short compared to normal sleepers (95%CI = 0.16–1.38, p = 0.01). When we introduced in the model the difference between weekend and weekday sleep duration as an additional variable, that may represent the degree of chronic lack of sleep sustained by the children (the more sleep is restricted during the week, the larger the weekend 'recovery' sleep response may be), thus trying to provide clues to the role of sleep restriction as opposed to absolute sleep duration as predictive of BMI, we did not find any significant statistical relationship (β = −0.09, 95%CI = −0.41–0.22, p = 0.55) (data not shown).

Then, we also modeled overweight/obesity as the outcome variable and we found a significant association with chronic lack of sleep both in univariate analysis (OR = 1.44; 95% CI = 1.01–2.06; p = 0.04) and after adjusting for children lifestyle habits (OR = 1.47; 95% CI = 1.01–2.12; p = 0.04) (data not shown).

## Discussion

The important new insights added by our study, concerning the association between short sleep and higher BMI, are related to the investigated age group and the objective classification of children's sleep duration by age-specific percentile curves.

Our study confirms, from a cross-sectional point of view and after adjustment for many factors influencing body weight, that chronic lack of sleep is associated with higher BMI in children around 10 years old. Several studies both cross-sectional [10]–[16] and longitudinal [17]–[23] have reached similar conclusions, some of them using overweight and obesity as the outcome variable, some measuring exposure as short/long duration of sleep with various cut-offs for number of hours of sleep [11], [12], [16], [20], [21] or as a continuous variable in number of hours of sleep [13]–[15], [17], [19], [22]. Moreover, different age groups have been taken into account as well as different methodologies in the ascertainment of hours of sleep (actigraphic measurements, diary reports, parental reports of children's sleep habits).

In regards to age, the data presented in this study provide useful insight on the relationship between insufficient sleep and BMI in a transitional stage of life, i.e. middle childhood towards adolescence. Indeed, numerous larger studies have been conducted in younger children [11], [12], [16], [18], [21], [22], whereas little is known about children with an average age of 10 years and, above all, the results in this age group are somewhat inconsistent. The few and small studies that have investigated this age have considered wider age ranges, e.g. 5/6–10 years [14], [24] or 10–20 years [25], while larger studies [16], [19] have explored the age between 3 and 17/18 years. The great interest to this transitional stage of life is related to the physical and psychological changes, typical of middle childhood, that influence sleep habits and pattern, such as the tendency to go to sleep and to wake-up later or to greatly modify weekend sleep schedules compared to weekdays.

As regards to measurement of sleep duration, differences in definitions of short sleepers varied substantially across studies, ranging from less than 8 hours [10], [15] to less than 12 hours per night [14]. This variability makes it very difficult to compare the different studies, reflects our current poor understanding of what the most effective measure scheme is and, finally, the large variability among children's sleep duration. It is necessary, therefore, to take into account the age-specific needs of the individual child and the use of percentile curves appeared to us the most appropriate tool to achieve this aim. In addition, we have chosen to use the sleep duration as a dichotomous variable rather than as a continuous, since we preferred to clearly define the population in which a significant effect of sleep duration on BMI can be expected.

As reported in previous studies using BMI as the outcome variable, the changes in BMI influenced by sleep duration do not seem so impressive as the ORs reported in studies using overweight/obesity as the outcome variable [22], [26]. However, we agree with those authors that changes in BMI are interesting from a public health point of view, since even small shifts of the BMI distribution have great impact at the population level [22], [26]. Anyway, we also modeled overweight/obesity as the outcome variable and we found a significant association with chronic lack of sleep.

Several hypothesis have been formulated on the basis of this association and it has been suggested that sleep exerts a control mechanism on the metabolic and endocrine systems and, mainly, on the ratio of ghrelin and leptin, of the profile of insulin, glucose [27] and, also, on the secretion of growth hormone (GH) [10]. Moreover, a recent study [28] on the brain activation in subjects with acute sleep deprivation in reaction to food stimuli has shown an increase of frontal cortex activity. This brain area has been found to have an higher activation in obese rather than in normal weight subjects [29].

We conducted our analysis taking into account several known factors influencing BMI. Previous studies have shown that overweight/obesity in parents, high birth weight, physical inactivity and poor eating habits are significantly related to childhood overweight and obesity, and our results are consistent with these studies [14], [16], [30].

Although this was not a main aim of our study, we found a high prevalence of short sleepers and, as expected, of overweight and obesity. Comparison with results of a survey conducted on the Italian population showed that our prevalence of overweight was aligned with that reported in the Calabria Region (26%) and higher than that in Italy (23%), while we found a lower prevalence of obesity, with a value of 7% versus 15% in Calabria and 11% in Italy [31]. Our lower obesity prevalence may be related to the age of the examined sample, which was on average older than that of the Italian monitoring system [31]. Furthermore, an international survey investigating health behaviour in school aged children has shown a tendency for older children to exhibit lower BMI with a reduction of obesity [32] and, in particular, in the area where our study was conducted, the prevalence of obesity was 4% in 11 and 2.2% in 15 years old children [33].

The mean total sleep duration reported in our study showed the presence of unhealthy sleeping habits in Italian children with 38.9% of children classified as short sleepers and a mean sleep duration of 9.4 (SD±0.6). Although comparisons with the results of previous survey on sleep behavior are difficult, since the measures adopted to evaluate sleep as well as data collection methods and sample characteristics were different, this result is consistent with values given by a multicentric study involving eight European countries on the distribution of sleep duration in children [34]. We found a later bedtime than that reported for children of the same age in different countries [7], with children included in our study who on average went to bed at 9∶55 p.m.( ±39 min), whereas our results are similar to those reported in a previous Italian survey [35].

### Limitations

As in most analogous research [14]–[16], [26], sleep duration in our study was obtained by parents self-reporting and there are no available sources to verify or validate this information. Although it is unlikely that this measure can provide a totally accurate account of sleep duration, it has been found that parental reports of sleep behaviours have considerable validity and reliability when compared with objective actigraphic measures [36], [37], even though parents of school-aged children are less accurate at identifying night waking in their child [37]. Therefore, information provided by parents, at worst, may have led to an underestimation of the prevalence of short-sleepers and, as a consequence, of the role of short-sleep duration on overweight and obesity. Finally, this method for detecting sleep behaviors in healthy children is non-invasive, cheap and can explore sleep for extended periods of time.

Another limit of this study is that we have not investigated the qualitative aspects of sleep disorders and the causes of the chronic lack of sleep as, for example, sleep latency, anxiety, daytime sleepiness, but these were not included among our aims.

Finally, since the study was cross-sectional in design, there was no temporal separation between explanatory variables and outcomes; therefore cause and effect relationships could not be determined. However our data have contributed to the improvement of the description of the complex link between sleep and obesity.

### Conclusion

In conclusion, our study showed that chronic lack of sleep appears to be associated with higher BMI even in middle childhood. Therefore, considering that, as in most western countries, we also found that overweight and obesity, unhealthy food and sleep habits were broadly diffused in our population, our results strongly suggest that public health strategies, focused on promoting healthy lifestyles should include an innovative approach to ensure an adequate duration of sleep at night especially in children, alongside more traditional approaches focused on balanced diet and regular physical activity. Routine paediatric evaluation of sleep pattern is also urged as well as the use of percentile curves to classify short, normal and long sleepers.
